# Replacement of the essential *Dictyostelium *Arp2 gene by its *Entamoeba *homologue using parasexual genetics

**DOI:** 10.1186/1471-2156-8-28

**Published:** 2007-06-06

**Authors:** Mehreen Zaki, Jason King, Klaus Fütterer, Robert H Insall

**Affiliations:** 1 School of Biosciences, University of Birmingham, Edgbaston, Birmingham B15 2TT, UK; 2 Cardiff University, Biomedical Sciences Building, Museum Avenue, PO box 911, Cardiff CF10 3US, Wales, UK

## Abstract

**Background:**

Cell motility is an essential feature of the pathogenesis and morbidity of amoebiasis caused by *Entamoeba histolytica*. As motility depends on cytoskeletal organisation and regulation, a study of the molecular components involved is key to a better understanding of amoebic pathogenesis. However, little is known about the physiological roles, interactions and regulation of the proteins of the *Entamoeba *cytoskeleton.

**Results:**

We have established a genetic strategy that uses parasexual genetics to allow essential *Dictyostelium discoideum *genes to be manipulated and replaced with modified or tagged homologues. Our results show that actin related protein 2 (Arp2) is essential for survival, but that the *Dictyostelium *protein can be complemented by *E. histolytica *Arp2, despite the presence of an insertion of 16 amino acids in an otherwise highly conserved protein. Replacement of endogenous Arp2 with *myc*-tagged *Entamoeba *or *Dictyostelium *Arp2 has no obvious effects on growth and the protein incorporates effectively into the Arp2/3 complex.

**Conclusion:**

We have established an effective two-step method for replacing genes that are required for survival. Our protocol will allow such genes to be studied far more easily, and also allows an unambiguous demonstration that particular genes are truly essential. In addition, cells in which the *Dictyostelium *Arp2 has been replaced by the *Entamoeba *protein are potential targets for drug screens.

## Background

*Entamoeba histolytica*, the causative agent of amoebiasis, is associated with significant morbidity and mortality worldwide [1]. The organism has a simple life cycle, existing either as an invasive trophozoite or as the infectious cyst. The highly motile trophozoites can be powerful pathogens and posses a host of virulence factors including an adherence lectin, pore forming proteins, extracellular-matrix cleaving proteases and impressive phagocytic capabilities [2]. The cytoskeleton and motility feature prominently in the parasite's motility, contact-dependant killing and phagocytosis of host cells [3,4] and as such a study of the molecular components involved is key to a better understanding of amoebic pathogenesis. While there have been substantial advances in the molecular biological tools available [5-7], *Entamoeba *remains genetically intractable, which has hindered full analysis of its lifestyle. Particular problems include the lack of a map of the genome [8,9], and failure to generate targeted gene disruptions.

In contrast, the amoebazoan *Dictyostelium discoideum*, an evolutionary relative [10], is an excellent model for the study of fundamental cellular processes such as cell motility, chemotaxis, phagocytosis and signal transduction [11]. That it is one of the easiest organisms to manipulate experimentally, is reflected in the ease with which genes can be disrupted, silenced, overexpressed and/or tagged [12]. *Dictyostelium *genes can be further manipulated using the technique of parasexual genetics [13,14]. The parasexual cycle provides a means of fusing two haploid strains together to form a relatively stable diploid containing both parental sets of chromosomes, which can then be segregated to make new haploids with reassorted chromosomes. This technique was widely used in the past [15,16]. More recently, this method has been made more powerful and flexible for use in conjunction with molecular genetic manipulation by being adapted for use in axenic conditions [17]. The ability to bring about non-sexual recombination of two different strains and subsequent re-segregation of recombinant haploid cells has several applications, in particular the crossing of pre-existing mutants to yield complex or multiple knockouts.

There is, however, a significant weakness in the *Dictyostelium *field, which concerns manipulation of essential genes. The combination of an organism that is generally haploid with a preference for full genetic knockouts has made essential genes hard to work with, and thus frequently under-studied. In addition, workers in the field have frequently asserted that genes are essential for growth when attempted knockouts fail, but the true cause is often experimental failure (for example *ras*D; [18,19]. Thompson & Bretscher [20] have described an elegant approach for making temperature sensitive targeted mutants, but a combination of laborious screening and narrow differences between permissive and restrictive temperatures has seen this technique rarely used. Conditional antisense and RNAi have been successful [21], but Rosel & Kimmel [21] could only achieve a 75% knockdown of mRNA and an unknown drop in protein levels; furthermore, the conditional phenotype was genetically unstable. Diploids, on the other hand, should allow single copies of genes to be disrupted and replaced using normal techniques. Genes can then be manipulated and replaced before the diploids are segregated, and then can be demonstrated to be essential if it is impossible to segregate disrupted haploids.

In this study, we have used parasexual genetics to manipulate the *E. histolytica *actin related protein 2 (Arp2) by gene replacement in the genetically-tractable *Dictyostelium*. Our results show that *Entamoeba *Arp2 complements analogous *Dictyostelium *mutations at all stages of the life cycle. In addition our results provide an unambiguous demonstration of the lethality of *Dictyostelium *Arp2 mutations.

## Results

### Conserved and unique aspects of *Entamoeba *Arp2

The Arp2/3 complex is central to the control of actin polymerisation [22]. The complex is composed of seven subunits, including the actin related proteins 2 and 3 and five other proteins. These proteins have been strikingly conserved throughout eukaryotic evolution [23,24]. Using *Dictyostelium *Arp2 [25], encoded by gene *arp*B, as the query we identified a single *Entamoeba *homologue in BLAST searches of the *Entamoeba *genome project [26]. As in *Dictyostelium *[25], there is no evidence of other isoforms. Unambiguous demonstration that there are no isoforms will require a more complete genome sequence than is currently published.

**Figure 1:**
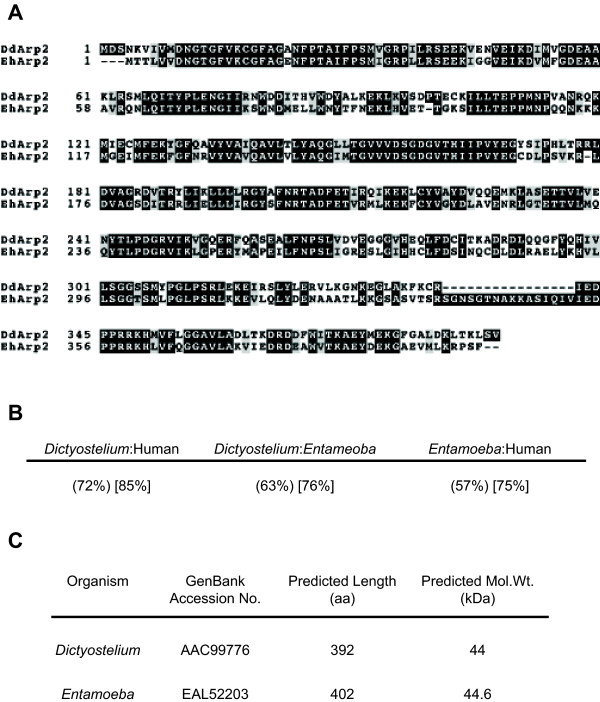
**Comparison of Arp2 from *Dictyostelium *and *Entamoeba***. **(A) **Sequence alignment of Arp2 from *D. discoideum *(Dd) and *E. histolytica *(Eh). Dark shading represents identical residues and residues present in any one specie. Light shading represents conserved residues. Dashes are incorporated to optimise alignment. **(B) **Comparison of *Dictyostelium *Arp2 with the human, *Saccharomyces *and *Entamoeba *homologues. Numbers in () show percent identity (the proportion of identical amino acids over the full length of the aligned proteins expressed as a percentage). Numbers in [] show percent similarity (the proportion of both identical and conserved amino acids over the full length of the aligned proteins expressed as a percentage). **(C) **Key features of Arp2 from *Dictyostelium *and *Entamoeba *based on respective GenBank entries.

A comparison of the *Dictyostelium *and *Entamoeba *Arp2 sequences is shown in Fig. 1. *Entamoeba *Arp2 shows substantial amino acid sequence homology to both *Dictyostelium *and human proteins, somewhat more to *Dictyostelium*. However, alignment of *Dictyostelium *and *Entamoeba *protein sequences reveals an unexpected insertion of approximately 16 amino acids between residues 337 and 352, which is apparently unique to *Entamoeba *(Fig. 1A). This led us to compare the *Entamoeba *sequence with its counterparts from other eukaryotes. Allowing for bias in the gaps introduced to optimise alignment, comparison of Arp2 from *Entamoeba*, *Dictyostelium*, Human, mouse, *Drosophila*, yeast and nematode shows that this region is unique to *Entamoeba *(see Additional File 1).

### Manipulation of essential genes using *Dictyostelium *parasexual genetics

Normal vegetative *Dictyostelium *grow as haploids. Occasionally however, two haploids can fuse together during normal growth to form a diploid containing both parental sets of chromosomes within a single nucleus. The diploid state is relatively stable, and cells grow and behave much the same as their haploid parents. Diploid cells may spontaneously lose one copy of each chromosome at random, thus reverting back to a haploid state with a selection of chromosomes from both parents. This 'parasexual' cycle has recently been adapted for use in axenic conditions [17].

**Figure 2:**
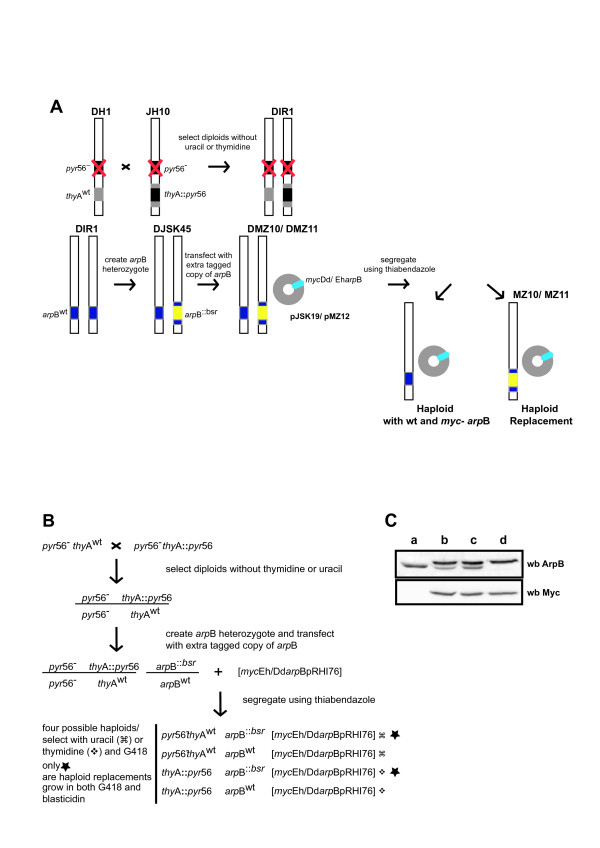
**Gene replacement using parasexual genetics**. **(A) **Schematic representation of the method. **(B) **A scheme showing the genetics underlying the gene replacement method. **(C) **Western hybridisation screen for the gene replacement method. Banding patterns seen with *Dictyostelium *anti-Arp2 and mAb 9E10 anti-*myc *antibody are shown. Lane 1 shows the pattern seen for strains DH1, JH10, DIR1 and DJK45; lane 2 shows the pattern seen for strains DMZ10 and DMZ11; lane 3 shows the pattern for a haploid strain containing both wild-type and *myc*-tagged Arp2; lane 4 shows the pattern of the replacement strains MZ10 and MZ11.

We have designed a scheme that allows us to manipulate and study essential genes by gene replacement. A diagrammatic representation of the scheme is shown in Fig. 2A, while Fig. 2B details the underlying genetics. Since diploid cells contain two copies of each gene, it is possible to knock out one of the copies to give a heterozygote. As this cell line still contains a wild-type copy of the gene, the mutation is complemented, making it possible to disrupt essential genes with no apparent phenotype. We can then introduce an additional copy of the gene on an extrachromosomal vector, with or without an affinity tag, such that on segregation we can select for cells carrying the disrupted chromosomal allele with the extrachromosomal copy as a gene replacement. For this scheme to work a varied/significant complement of selection markers is needed, allowing identification of both haploid parents used to create the diploid cell line, the disrupted chromosomal allele and the replacement gene copy. This is achieved by combining multiple nutritional (auxotrophic) and drug selections (Fig. 2A and 2B).

### Arp2 heterozygotes are normal

The *Dictyostelium *DIR1 cell line [17] is a fusion of haploid parents DH1 (uracil auxotroph) and JH10 (thymidine auxotroph) (Table 1). It can thus be maintained in a diploid state in minimal medium or HL-5 without added uracil or thymidine (Table 2). We disrupted one of the two copies of Arp2 using a cDNA with a *Bsr *gene inserted in the *Bst*BI site (see Additional File 2A). Successful disruptants were identified using a PCR screen with one primer targetted inside the *Bsr *gene and another outside the transfected construct, so only successful integration of the construct by homologous recombination would generate a product (see Additional File 2B). The resulting mutant, named DJK45, was straightforwardly generated, even though Arp2 was thought to be essential – we had previously not been able to disrupt the gene in haploids despite exhaustive attempts. Like DIR1, the diploid Arp2 heterozygote DJK45 is maintained in normal growth medium (Table 2). DJK45 cells grow at apparently normal rates, migrate normally and develop with normal timing (data not shown).

**Table 1:** Cell lines used in this work.

**Strain**	**Parent(s)**	**Description**	**Genotype**	**Ref.**
HM-1:IMSS (clone 9)	HM-1:IMSS	*E. histolytica *Lab strain	Wild type	[49]
AX3	NC4	*D. discoideum *Lab strain	Wild type	[44]
DH1	AX3	Uracil auxotroph	*pyr*56Δ*thy*A^wt^	[50]
JH10	AX3	Thymidine auxotroph	*pyr*56^- ^*thy*A::*pyr*56	[51]
DIR1	DH1/JH10	Diploid between DH1 & JH10 (*thy*A and *pyr*56 alleles complemented – no dependence; Arp2 homozygote)	*pyr*56^-/Δ ^*thy*A::*pyr*56/^wt^	[17]
DJK45	DIR1	Arp2 heterozygous diploid (*bsr*^r ^inserted in one copy of Arp2)	*pyr*56^-/Δ ^*thy*A::*pyr*56/^wt ^*arp*B^-*bsr*/*wt*^	This work
DMZ10	DJK45 + pJSK19	Arp2 heterozygote expressing *myc*- tagged replacement *Dictyostelium *Arp2	pyr56^-/Δ ^thyA::pyr56/^wt ^*arp*B^-*bsr*/*wt *^+ [*myc*Dd*arp*BpRHI76]	This work
DMZ11	DJK45 + pMZ12	Arp2 heterozygote expressing *myc*- tagged replacement *Entamoeba *Arp2	*pyr*56^-/Δ ^*thy*A::*pyr*56/^wt ^*arp*B^-*bsr*/*wt *^+ [*myc*Eh*arp*BpRHI76]	This work
MZ10	DMZ10	Thymidine dependent haploid rescued with replacement *Dictyostelium *Arp2	*pyr*56^- ^*thy*A::*pyr*56 *arp*B^-*bsr *^+ [*myc*Dd*arp*BpRHI76]	This work
MZ11	DMZ11	Thymidine dependent haploid rescued with replacement *Entamoeba *Arp2	*pyr*56^- ^*thy*A::*pyr*56 *arp*B^-*bsr *^+ [*myc*Eh*arp*BpRHI76]	This work

**Table 2:** Growth conditions of cell lines used in this work.

**Strain**	**Growth Conditions**
HM-1:IMSS (clone 9)	Axenic medium LYI-S-2
AX3	Axenic medium HL-5
DH1	HL-5 supplemented with Uracil
JH10	HL-5 supplemented with Thymidine
DIR1	HL-5
DJK45	HL-5 (blasticidin optional)
DMZ10	HL-5 supplemented with G418 (blasticidin optional)
DMZ11	HL-5 supplemented with G418 (blasticidin optional)
MZ10	HL-5 supplemented with Thymidine and G418 (blasticidin optional)
MZ11	HL-5 supplemented with Thymidine and G418 (blasticidin optional)

### Superior segregation of haploids from diploids on bacterial lawns

Diploids will, over time, segregate to give haploids with all possible combinations of parental chromosomes. Our diploid strains are, however, very stable, so spontaneous segregation occurs at low enough rates to make the method impractical for experimental use. The segregation process can be accelerated, by treating diploids with microtubule inhibitors such as benlate (benomyl) and thiabendazole [27,28]. Thiabendazole, which has proved the easiest to work with in both axenic conditions and on bacterial plates due to its greater solubility in aqueous solutions, has been used in this study.

Table 3 summarises the results achieved with both methods. It is immediately evident that segregation is more efficient on bacterial plates than the axenic protocol. Following segregation in axenic medium, a total of 152 clones were screened over the course of three separate experiments, while 36 clones were screened from two independent experiments using bacterial plates. Segregation on bacterial plates resulted in approximately 8 times more haploid cell lines than in axenic medium.

**Table 3:** Segregation efficiency in axenic medium and on bacterial plates.

**Segregation Conditions**	**Proportion Diploid**	**Proportion Haploid**	**Proportion Haploid Replacement**
Axenic	147/152 (96.7%)	5/152 (3.3%)	2/152 (1.3%) [2/5 (40%)]
Bacterial Plates	3/36 (8.3%)	10/36 (27.8%)	6/36 (16.7%) [6/10 (60%)]

Cells were segregated in axenic medium as described in Materials and Methods. Numbers are representative of three experiments.

Cells were segregated on bacterial plates as described in Materials and Methods. Numbers are representative of two experiments.

Numbers represent all haploids i.e. haploids with extra tagged copy of Arp2 and haploid replacements.

Numbers reflect haploid replacements expressed as a proportion of the total number of haploids ().

Several attempts were made in an effort to improve efficiency of the axenic medium protocol with no observable difference (data not shown). Lower concentrations of thiabendazole (2 μg/ml), which is the working dose in bacterial plates, did not result in any haploids while higher doses were lethal. The protocol as stated here and previously [14] calls for diploid cells to be incubated for three days in the presence of a single dose of 5 μg/ml of thiabendazole, followed by three days of recovery. Experiments were carried out where 5 μg/ml of thiabendazole was added per day for three successive days followed by recovery as before, with no significant difference. Altering the number of days of recovery had no visible effect.

### Arp2 is essential for *Dictyostelium *survival

An advantage of using diploid strains is that they can be used as tools to manipulate and study genes essential for cell survival. Diploid heterozygous cell lines can be used to test the lethality of a mutation; if a particular gene mutation were lethal, segregation would result in haploid progeny containing the wild-type allele only. In this vein, segregation of the diploid Arp2 heterozygous cell line DJK45 alone resulted in haploids containing only wild-type Arp2. We never managed to isolate a blasticidin resistant cell line from a total of 98 thiabendazole-treated clones screened over the course of two independent experiments. Likewise, segregation of DJK45 cells transfected with the empty vector pRHI76 did not result in any blasticidin resistant haploids. 116 clones were screened in three independent experiments and the resulting haploids were all blasticidin sensitive, and therefore only contained the undisrupted *arp*B gene. Taken together these results demonstrate that Arp2 appears to be completely essential for *Dictyostelium *cell survival. This is unlike *Saccharomyces *[29], in which Arp2 gene disruption reduces, but does not completely abrogate cell growth.

### Generation of strains containing *arp*B gene replacements

**Figure 3:**
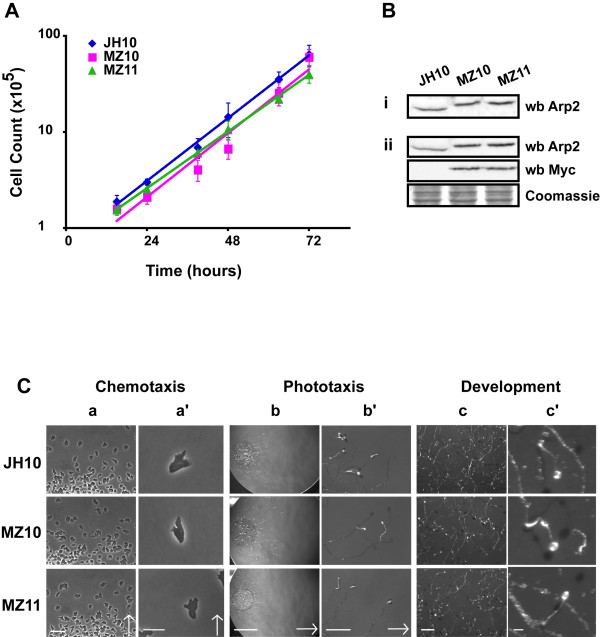
**Phenotypic analysis of replacement clones**. **(A) **Growth in HL-5 medium in shaken flasks. **(B) **Protein expression in vegetative cells; (i) Cell lysates prepared from equal numbers of cells were separated by SDS-PAGE, transferred onto nitrocellulose and immunoblotted as described in Materials and Methods. Blots were probed with *Dictyostelium *anti-Arp2 antibody. (ii) 20 μg of total protein was separated by SDS-PAGE, transferred onto PVDF and immunoblotted as described in Materials and Methods. Blots were probed with *Dictyostelium *anti-Arp2 and mAb 9E10 anti-*myc *antibodies. **(C) **All results are representative two to three experiments. **Chemotaxis**. Under-agar migratory response of cells to a gradient of folate. Arrows indicate the direction of cell migration (increasing folate concentration); **(a) **Scale bar is 50 μm. **(a') **Scale bar is 10 μm. **Phototaxis. **Response of cells spotted on nitrocellulose filters, to a lateral light source (48 hrs). The light source is always to the right of the image. Arrows indicate the direction of migration; **(b) **Scale bar is 5 mm. **(b') **Scale bar is 1 mm. **Development. **Fruiting body morphology of cells after development on nitrocellulose filters (48 hrs). **(c) **Scale bar is 1 mm. **(c') **Scale bar is 0.1 mm.

The diploid Arp2 heterozygote, DJK45, was used to generate the DMZ11 cell line which carries an extra tagged copy of *Entamoeba *Arp2 (Table 1; Fig. 2A and 2B). To introduce the extra copy a *myc*-tagged *Entamoeba *Arp2 construct was cloned into the *Bgl*II-*Not*1 sites of the extrachromosomal expression vector pRHI76 and the plasmid (pMZ12) was electroporated into DJK45 cells. The cloning site in pRHI76 is located downstream of the constitutive actin 15 promoter which drives expression of the *myc*-Arp2. A *neo*^r ^gene allowed transformants to be selected with G418 (Table 2). DMZ10 cells carrying an extra *myc*-tagged copy of *Dictyostelium *Arp2 (pJSK19) were generated using the same method (Table 1; Fig. 2A and 2B). By comparing the different *myc*-tagged Arp2 transformants probed with anti-Arp2 and anti-*myc*, we were able to show that our rabbit anti-Arp2 antibody recognised *Entamoeba *and *Dictyostelium *isologues with approximately equal efficiency (Fig. 3B).

Both DMZ10 and DMZ11 cell lines were segregated in axenic medium as well as on bacterial plates as before. As observed with simple segregation, the proportion of haploid replacements resulting from bacterial plates is also very much higher (approximately 13-fold in these cases). When the number of haploid replacements is considered as a proportion of the total number of haploids, the difference between the two methods of segregation though still significant is not as marked (1.5 times more bacterial haploids contain replacements), though this difference is experimentally irrelevant in the light of the low efficiency of axenic segregation.

The method for segregation on bacterial plates, though clearly efficient, is not without some limitations. Following the method as described here, the cells spend a significant period of time (approx. 3 weeks) in the absence of any/all nutritional and drug selections. This is a cause for concern – 23 of the 36 clones (63.9%) screened following segregation on bacterial plates did not grow in any of the axenic selection conditions used for screening. We believe this may be caused by loss of the extrachromosomal plasmids (pMZ12 and pJSK19), yielding cells that are no longer G418 resistant. Segregation in axenic medium, though inefficient, enables use of all desired selections throughout the procedure, so the plasmids cannot be lost in this way.

Notwithstanding the segregation efficiency, we have successfully used the methods and scheme described here to generate *Dictyostelium *cell lines carrying *myc*-tagged copies of the *Entamoeba *and *Dictyostelium *Arp2 genes as replacements of the endogenous, disrupted copies (Table 1; Fig. 2A and 2B). This further confirms that Arp2 is essential for Dictyostelium growth. An empty vector does not allow haploids to be recovered, but a vector that drives Arp2 expression does. Segregation of DMZ10 and DMZ11 resulted in the isolation of two haploid replacement clones each. As mentioned above, haploid segregants from DIR1 must be auxotrophic for either uracil or thymidine; the four replacement clones isolated by us were all thymidine dependent (Table 1; Fig 2B and data not shown). This result suggests a bias against the *pyr*56 null allele. We believe this is due to the large deletion surrounding the *pyr*56 locus that was made when the DH1 line was created. The intention had been to completely remove *pyr*56 sequences, but it appears that genes either side of *pyr*56 were also affected, leading to an overall loss of fitness. Preliminary analysis revealed no obvious differences between each clone from the same diploid parent. One clone of each type was named (MZ10 for the *myc*-tagged *Dictyostelium *gene and MZ11 for the *Entamoeba*; Table 1), and used for further analysis.

### Screen for haploid replacements

In our previous work [17], cell ploidy was verified using fluorescence activated cell sorting (FACS) and chromosome staining. In this case we were able to use the presence of the *myc*-tag to design a western blotting based screening method. Fig. 2C shows a typical screen. Cell lines containing only a wild-type Arp2 give a single band at about 45 kDa with the *Dictyostelium *anti-Arp2 antibody (Fig. 2C lane 1). These include the haploid parents JH10 and DH1, the diploid DIR1 and the diploid Arp2 heterozygote DJK45 (Table 1). Cells with an extra, *myc*-tagged copy of Arp2 show two bands, the lower from the wild-type allele and the upper (approx. 47 kDa) the *myc*-tagged copy (Fig. 2C lanes 2 & 3). Cell lines displaying this pattern include DMZ10 and DMZ11 cell lines (Table 1; Fig. 2A and 2B) and haploid cells which after segregation carry both the wild-type allele and the extra *myc*-tagged copy (Fig. 2A and 2B). Finally, haploid replacement clones, which contain the extra *myc*-tagged Arp2 only, give a single band (Fig. 2C lane 4) which coincides with the upper bands seen in lanes 1, 2 and 3 (Fig. 2C). The anti-*myc *mAb 9E10 reveals a single band each in all cell lines carrying the extra, tagged copy of the Arp2 (Fig. 2C). This band coincides with the upper bands seen with the anti-Arp2 antibody.

### *Entamoeba *Arp2 complements analogous *Dictyostelium *mutations

Determination of the vegetative growth rate in shaken suspension showed that the *Entamoeba *Arp2 replacement (MZ11) cells grow at slightly slower rates than the equivalent parent (doubling time of 12 hrs compared with 11 hrs respectively; Fig. 3A). Cells in which Arp2 was replaced by *myc*-tagged *Dictyostelium *Arp2 (MZ10) grew at the same rate as the parent. Levels of Arp2 protein were determined by western blotting using an antibody raised against *Dictyostelium *Arp2. Levels of Arp2 protein in vegetative *Entamoeba *replacement cell lines MZ11 are similar to those of the *Dictyostelium *replacement MZ10 cells (Figs. 3Bi &3Bii). Both replacements contained somewhat higher Arp2 levels than the parental cells.

MZ11 cells chemotax towards folate and on starvation aggregate to form motile phototactic slugs, and differentiate into morphologically normal fruiting bodies with a time course comparable to the parent JH10 and the *Dictyostelium *replacement MZ10 cells (Fig. 3C). *Dictyostelium *has an unusual life cycle. On starvation, amoebae chemotax towards one another and aggregate to form multicellular structures. One such structure, the slug, is strongly phototactic (migrates towards light). Aggregates eventually develop into a fruiting body, which is composed of a differentiated spore mass carried atop a stalk. *Dictyostelium *development provides an insight into several aspects of cellular biology such as cell locomotion, cell-cell interactions and signal transduction. Multiple different mutants in these processes [30,31] show obvious effects on development and phototaxis. Using these and similar assays, we find that *Entamoeba *Arp2 complements the analogous *Dictyostelium *mutations at every stage of the uni-and multi-cellular life cycle (Fig. 3).

**Figure 4:**
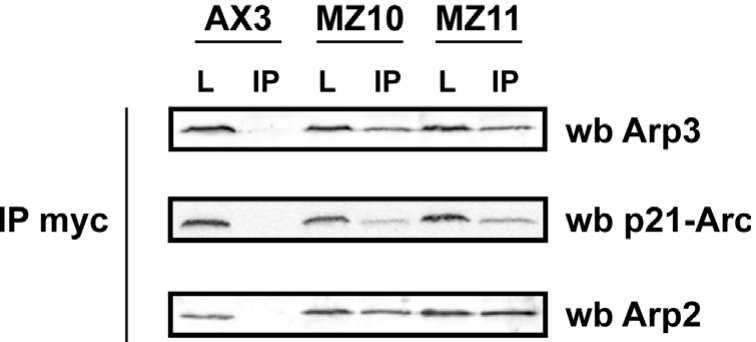
**Immunoprecipitation**. Washed, pelleted beads with bound mAb 9E10 and immunoprecipitated proteins were analysed by SDS-PAGE and western blotting as described in Materials and Methods. Cell lysates (L) and immunoprecipitates (IP) from three *Dictyostelium *cell types were examined; AX3 (wild type), MZ10 (haploid rescued with *myc*-tagged *Dictyostelium *Arp2 replacement), MZ11 (haploid rescued with *myc*-tagged *Entamoeba *Arp2 replacement). Blots were probed with antibodies to *Dictyostelium *Arp2, Arp3 and the p21-arc subunits.

Together these results present convincing evidence that *Entamoeba *Arp2 is a fully effective replacement for *Dictyostelium *Arp2. We would therefore, expect the *Entamoeba *Arp2 to exist as a part of the large seven member *Dictyostelium *Arp2/3 complex. To confirm this cell extracts from both the *Dictyostelium *and *Entamoeba *replacement cell lines MZ10 and MZ11 and the parent AX3 were immunoprecipitated with mAb 9E10 directed against the *myc *epitope. The precipitates were analysed by western blotting with antibodies directed to the *Dictyostelium *Arp2, Arp3 and the p21-arc subunits. The *myc *antibody pulls down all three Arp2/3 complex members in MZ10 and MZ11 cell lines, which contain the *myc*-tagged copy of Arp2 as a gene replacement (Fig. 4). Using the *myc *antibody on the untransformed wild type strain Ax3 does not pull down any of the complex members. As expected, when the experiment is done in reverse and cell extracts are incubated with antibody directed to Arp2 followed by western analysis with the anti-*myc *antibody, protein bands are only seen with the MZ10 and MZ11 cell lines (data not shown). The *Entamoeba *replacement Arp2 is therefore being incorporated into the *Dictyostelium *Arp2/3 complex.

## Discussion

*E. histolytica *is a significant cause of worldwide disease [1]. Progress has been made in identifying genes and proteins related to its virulence [2] and sequencing of the genome [8] will make molecular identification even easier. There is now a need to characterise the physiological role of these molecules. A key limitation to studying and understanding *Entamoeba *biology has been the lack of effective methods for manipulating genes. This parasite has proven resistant to most classical genetic techniques, homologous recombination has not yet been used and at present gene function analysis is limited to the expression of mutant genes [5] or by gene silencing [6,7]. We describe here a method for studying *Entamoeba *genes using *Dictyostelium*, a close evolutionary relative [10].

We have used *Dictyostelium *parasexual genetics to develop a scheme that allows us to study *Entamoeba *genes by replacement. The method works in both axenic medium conditions and on bacterial plates and the replacement clones can be easily screened for and identified using simple western blotting. The procedure, from segregation to final isolation of a replacement clone, takes a little over a month with only a few concentrated periods of hands-on work in that time. One limitation is that intrachromosomal crossovers are at present not possible, so only whole chromosomes can be recombined. We should however, be able to address this in the future as mitotic recombination and sexual cross over are both known to occur in *Dictyostelium *[32,33].

In our experience, segregation on bacterial plates proved far more efficient than in axenic medium. This could be because conditions provided by the plates are kinder to cells and help aneuploids to survive, giving them more time to resolve into true haploids. Thus, one way of optimising efficiency would be to use a combination of both methods described here. Cells could be exposed to thiabendazole and recovered in selective axenic medium before being cloned on bacterial plates. However, as observed by us, one limitation of cloning on plates is that a large proportion of the clones screened thereafter did not grow in any of the axenic selection conditions used for screening. One possible way of circumventing this would be to use selections, for example G418 [34], or blasticidin [35] in the plates. Alternatively, cells segregated on thiabendazole plates could be transferred directly to axenic medium containing all the appropriate selections, without the additional subcloning step (see Materials and Methods and [14]. This would reduce the time spent on plates without adequate selection and could thus further improve efficiency. After this primary screen, all correct replacement clone(s) must however be subcloned to ensure that they do not contain a mixed population [17]. A third possibility would be the generation of improved extrachromosomal plasmids; current Ddp1-based plasmids have been seriously rearranged during the generation of pATANB [36], and plasmids with more intact Ddp1 material would be expected to propagate more efficiently and be lost more slowly during bacterial growth.

*Dictyostelium *cells are normally haploid. While this facilitates isolation and identification of mutant phenotypes, it makes study of essential genes difficult. Our two-step gene replacement described here offers multiple advantages. Genes necessary for cell survival can be replaced with tagged or mutated genes, or even exogenous genes like the *Entamoeba *Arp2 we describe here. Single-crossover methods such as that described by Thompson and Bretscher [20] also allow replacement of essential genes, but the two techniques have contrasting advantages. Thompson's method is far better for isolating temperature-sensitive mutants, but ours is far easier for generating complete replacements of genes, as the Thompson method gives rearrangements in one end of a gene (usually the 5' end), not the complete gene as described in this work. In addition, if multiple modified versions of a gene (for example a series of point mutants) need to be used, our method requires only one homologous recombination to make the heterozygote. All subsequent steps are easier, quicker and require much less detailed analysis (the western blotting strategy shown here, for example, is quite unambiguous).

We also provide a new mechanism for experimentally demonstrating that genes are essential for growth. The *Dictyostelium *literature has been greatly complicated by assertions that genes are essential, which are in reality caused by experimental failure to generate a knockout. By disrupting genes in diploids we separate the homologous recombination from the generation of the final knockout – failure to segregate a haploid from a heterozygote is a far more thorough demonstration than failure to make a knockout in a haploid parent.

Using a diploid Arp2 heterozygous cell line we have shown that mutations in *Dictyostelium *Arp2 are lethal. Studies in numerous organisms have demonstrated the importance of the Arp2/3 complex in actin dynamics and cell viability [22]. The complex seems to be essential in most eukaryotes, though *Saccharomyces cerevisiae *can marginally survive deletion of a subset of the subunits [29], while in other yeasts such as *Schizosaccharomyces pombe *all seven Arp2/3 complex subunits are essential for viability [37,38].

Little if anything is known about the Arp2/3 complex in *Entamoeba*. We have used the *Dictyostelium *parasexual genetic scheme described here to allow future detailed studies of *Entamoeba *Arp2. Preliminary characterisation shows that *Entamoeba *Arp2 shares significant homology with the *Dictyostelium *counterpart in function as well as sequence. Our results from both functional assays and immunoprecipitation indicate that the amino acid residues necessary for interaction with ATP within actin and the other members of the complex are conserved [23]. Furthermore, presence of the *Entamoeba *Arp2 does not seem to harm the ability of *Dictyostelium *cells to respond to signals cues relayed by other cells or the environment.

The only obvious difference between the *Dictyostelium *and *Entamoeba *Arp2 was the presence of a 16 amino acid long insertion present only in *Entameoba*. The fact that this region is unique to *Entamoeba *when compared to all other model organisms, including humans, raises the possibility that this might be a potential drug target. The cornerstone of treatment and control for amoebiais is nitroimidazoles, mainly metronidazole [39]. To date there is no report of resistance to metronidazole. However, this drug is now used both therapeutically and prophylactically for numerous ailments, thus ensuring exposure of *Entamoeba *to it, and providing just the conditions under which drug resistance can develop [40]. *In vitro *resistance has been induced to metronidazole in two axenic *E. histolytica *strains [41]. New targets for new drugs are therefore increasingly needed.

The *Entamoeba *genome sequencing project has given evidence of significant horizontal gene transfer [8]. Furthermore, despite their being no confirmed evidence of sex in *Entamoeba*, the sequenced genome reveals an almost complete complement of genes required for meiosis [42]. In light of this it is unclear why it is so difficult to achieve homologous recombination in this parasite. We believe, that until classical methods such as gene knock-out and random integration can be achieved, the strategy described here will add to the methods already available in providing an effective tool for the study of gene function analysis in this organism. We hope that this method will be useful in analysing the functions and binding partners of other conserved proteins, especially those involved in virulence, for defining components and mechanisms of cell movement involved in metastasis and for identifying other novel protein/drug targets.

## Conclusion

We have further extended the use of axenic diploid cells for *Dictyostelium *genetics. We have shown that diploids can be used to demonstrate that genes (in this case one encoding Arp2) are essential. We have also shown that diploids heterozygote for essential genes appear normal, and that these heterozygotes can be segregated to give haploids with gene replacements.

We anticipate that *Dictyostelium *with *Entamoeba *Arp2, and similar replacements, will make screens for amoebicidal drugs more practical.

## Methods

### Cell culture

Axenic *E. histolytica *strain HM-1:IMSS clone 9 was maintained in LYI-S-2 medium [43], supplemented with 15% heat-inactivated adult bovine serum (Sigma-Aldrich), at 36°C. Unless otherwise stated, *Dictyostelium *cells were cultured axenically in HL-5 medium [44], supplemented with a cocktail of vitamins (20 μg/l biotin, 5 μg/l cyanocobalamin, 0.2 mg/l folic acid, 0.4 mg/l lipoic acid, 0.5 mg/l riboflavin, 0.6 mg/l thiamine) at 21/22°C either in petri plates or in shaken flasks. In some instances, cells were grown on SM agar plates in association with *Klebsiella aerogenes*. When screening for nutritionally dependant (auxotrophic) growth, either 20 μg/ml uracil or 100 μg/ml thymidine were added to the culture medium. For selections with G418 and/or blasticidin we used medium containing 10 μg/ml of the drugs. Genotypes of the cell lines used in this study are given in Table 1 and their growth conditions in Table 2.

### DNA extraction

*E. histolytica *genomic DNA was isolated as previously described [45,46], dissolved in 10 mM Tris-Cl, ImM EDTA (pH8) and passed over a Microspin S-200 HR column (Amersham Biosciences). RNA was removed by the addition of RNase A (Promega) to 0.05 mg/ml.

*Dictyostelium *genomic DNA was extracted as follows, cell pellets were washed three times in RLB buffer (0.32 M sucrose, 10 mM Tris-HCL, 5 mM MgCl_2_, 1% Triton X100), before incubation in 10 mM Tris-HCL pH 7.5, 5 mM EDTA, 0.35% SDS and 50 μg/ml proteinase K at 65°C for 30 minutes and then at 37°C for one hour. DNA was then purified using standard glassmilk protocols and eluted into water.

### Generation of the Diploid Arp2 heterozygote and replacement Arp2 vector constructs

Diploid heterozygous Arp2 cells (DJK45) were created using the Diploid DIR1 cell line as parent (Table 1 and [17]). A knockout construct (see Additional File 2) was generated from the cloned cDNA in the pSPORT cloning vector (plasmid FC-AH01). To facilitate PCR screening, the 5-prime end was truncated by digesting with *Sma*I/*Bsr*GI, treating with klenow and then re-ligating. The blasticidin selection cassette was then purified as a *Cla*I fragment from pRHI148, and ligated into the *Bst*BI site of Arp2. This was then excised by a *Kpn*I/*Not*I digest prior to transformation into *Dictyostelium*. Clones were screened by PCR using the primers CATTGTAAATTCGATAATAAGGG and ATAAAGCATTGTAATCTTCTCTG, which only give a product of 1081 bp upon homologous recombination.

The *E. histolytica *Arp2 homologue was identified by a BLAST search against the *Entamoeba *genome project [26] using the *Dictyostelium *Arp2 protein sequence as template (Fig. 1). The full length CDS sequence was amplified using Phusion high-fidelity DNA polymerase (Finnzymes, New England Biolabs, Inc) with a 5-prime *myc*-tagged primer containing a *Bam*H1 site (F- AGGGATCCAATAAAATGGAACAAAAATTAATTTCAGAAGAAGAT

TAATGACCACCTTAGTAGTAGAC) and a 3-prime primer containing a *Not*1 site (R- CTGCGGCCGATTAGAAACTAGGTCTCTTCAAC). The resultant PCR product was cloned using the Zero Blunt™ TOPO™ PCR cloning kit (Invitrogen™) and re-sequenced for verification. Finally, the *Bam*HI-*Not*1 amplification product was inserted into the extrachromosomal expression vector pRHI76, downstream of the constitutive actin 15 promoter, which drives expression of the *myc*-tagged *Entamoeba *replacement Arp2. *Myc*-tagged *Dictyostelium *replacement Arp2 constructs were made by PCR from a full-length cDNA clone as described above using a 5-prime *myc*-tagged primer (GAAGGATCCTAAAAAATGGAACAAAAATTAATTTCA) and a 3-prime primer containing an *Mlu*1 site (GAAACGCGTTTCATTTAAACAGATAATTTAG) and subcloning the amplification product into TOPO Blunt II (Invitrogen). This was then excised as a *Bam*HI-*Not*I fragment and reinserted into the pRHI76 expression vector as above. The resultant plasmid DNAs were electroporated into the *Dictyostelium *Arp2 heterozygous DJK45 cells as described below. Transformants were selected using G418 resistance.

### Transformation

Transformation of *Dictyostelium *cells was performed by a modification of Howard *et al *[47]. Briefly, exponentially growing cells (approx. 1.6 × 10^7 ^per zap) resuspended in electroporation buffer (10 mM potassium phosphate buffer pH6.1, 50 mM sucrose) were mixed with 10 μg of linearized DNA and electroporated in a BioRad gene pulser at 1.0–1.2 kV, 3 mF with a 5-ohm resistance in series. Following a 10 min incubation on ice, the cells were incubated for 15 min at 21/22°C with 5 ml of healing solution (100 mM CaCl2, 100 mM MgCl2) and then culture medium was added. Drugs for selection were added 24 hrs after electroporation.

### Segregation of Diploids

Diploid *Dictyostelium *cells were segregated in both axenic medium and on bacterial lawns as described by King and Insall [14].

Briefly, cells (2–2.5 × 10^7^) were segregated in axenic medium supplemented with 5 μg/ml thiabendazole as well as all the drug selections and nutritional additions required for the desired haploids. After three days, cells were recovered in axenic medium containing all the selections and nutritional supplements, but no microtubule inhibitors. Following three days of recovery, cells were plated clonally in axenic medium containing appropriate drug and nutritional selections and screened for the desired haploid/replacement.

Alternatively, clonally plated cells were segregated on bacterial plates supplemented with 2 μg/ml thiabendazole. After about two weeks cells from the edge of the colonies were subcloned on to bacterial plates without microtubule inhibitors. Approximately one week later samples from the edge of the colonies were picked and screened for the desired haploid/replacement in axenic medium containing appropriate drug and nutritional selections.

### Protein isolation and western analysis

To determine protein levels in cell extracts, cells were lysed by resuspension in SDS-PAGE sample buffer and boiled for 5 min. Samples (either the equivalent of approx. 5 × 10^6^/per lane or 20–25 μg total protein/lane) were subjected to SDS-PAGE and the separated proteins were transferred onto nitrocellulose membranes or PVDF (Amersham Biosciences). Blots were blocked with phosphate buffered saline (PBS)-Tween (0.1%) containing 5% nonfat dry milk for at least 1 hr at room tempt. Blots were incubated with antibodies directed to *Dictyostelium *Arp2 and Arp3 [25]Insall et al, 2001] and p21-arc (this work, see below) subunits, or mAb 9E10 anti-*myc *antibody (Cancer Research, UK). The bound antibodies were detected with secondary conjugated goat anti-rabbit and/or rabbit anti-mouse antibodies (Jackson ImmunoResearch Laboratories. Inc.). The bound secondary was detected using chemiluminescence (Pierce).

### Immunoprecipitation

Cell lysates were prepared by resuspending 3 × 10^7 ^cells in 1 ml lysis buffer (50 mM Tris (pH8.0), 100 mM NaCl, 1 mM EDTA, 1% Triton X-100 and protease inhibitors (1 mM PMSF, 1 mM TLCK) followed by gentle rotation and incubation at 4°C. The lysate was centrifuged and the supernatant was pre-cleared by incubation with protein G-sepharose beads (Cancer Research, UK) for 1 hr at 4°C with gentle rotation. Beads were removed by gentle centrifugation and the cleared lysate was incubated with mAb 9E10 anti-*myc *antibody bound to protein G-sepharose beads for 1 hr at 4°C with gentle rotation. Beads with bound mAb 9E10 and immunoprecipitated proteins were sedimented by gentle centrifugation and washed 4 times in lysate buffer. The washed pelleted beads were boiled in SDS-PAGE sample buffer and the supernatant was analysed by SDS-PAGE and western blotting as described above.

### Antibody generation

*E. coli *BL21(DE3) cells were transformed using plasmid pGEX2C-p21, encoding the p21 subunit of the Arp2/3 complex of *D. discoidium*, using heat shock. Overnight shaken cultures (37°C) containing 50 μg/ml ampicllin and 33 μg/ml chloramphenicol in Luria-Bertani broth (Sigma) were used to inoculate bulk cultures (2 × 1 l, 50 μg/ml ampicllin) and grown in shaken to an optical density of 0.25 (OD600) at 37°C, before being transferred to 25°C, equilibrated for 20 min, and then induced using 0.5 mM IPTG (final) for approx. 16 hours.

Cells were harvested by centrifugation, washed in PBS, and resuspended in 8:40 (v/v) mix of buffers GTLB2 (50 mM Tris-HCl pH 8, 100 mM MgCl2, 0.2% (w/v) Triton X-100, 0.02% (w/v) NaN_3_) and GTLB1 (50 mM Tris-HCl pH 8, 40 mM EDTA, 2.5% (w/v) sucrose, 0.02% (w/v) NaN_3_) + 1 mM PMSF. Cells were lysed by sonication and clear lysates obtained by centrifugtion at 21,000 rpm for 30 min at 4°C, were recycled for 2 hours over a pre-equilibrated glutathione-agarose column. Non-specifically bound protein was washed off using PBS containing 1 mM DTT and 0.2% Tween, followed by equilibration with TRB buffer (50 mM Tris-HCl pH 8, 5 mM MgCl_2_, 150 mM NaCl, 2.5 mM CaCl_2_, 1 mM DTT). The fusion protein was cleaved by addition of 10 units of thrombin to the column matrix and incubation for 6 hours. Cleaved p21 protein was eluted from the column, concentrated and further purified by size exclusion chromatography (sephacryl S-200 matrix, 300 ml column volume, 0.5 ml/min). Clean fractions were identified by SDS gel electrophoresis. Polyclonal antiserum was generated commercially using the standard rabbit immunization package from Sigma.

### Under agar chemotaxis assay

The response of cells to a folate stimulus was studied using the previously described under agar chemotaxis assay [48]. Briefly, three 2 mm wide troughs were cut 5 mm apart (4 cm length) in thin layers of 1% SM agar in Petri dishes. 200 μl of 0.1 mM folic acid or SM medium were added to the centre trough and allowed to form a gradient for around 1 hour at room temperature. 200 μl of about 1 × 10^6 ^axenically grown amoebae/ml suspended in SM medium were added to the peripheral troughs either side of the folic acid. Over the next 4–6 hrs cells were imaged as they moved under the agar towards the folate stimulus using phase contrast microscopy.

### Phototaxis

Qualitative phototaxis analysis was carried out as follows. Exponentially grown axenic cells (1 × 10^7^) were washed 1–2 times in KK2 (16.5 mM KH_2_PO_4_, 3.8 Mm K_2_HPO_4 _at Ph 6.0) and spotted onto nitrocellulose filters (Millipore), which had been soaked in the same buffer. Plates were wrapped in foil and incubated at 21/22°C with a lateral point light source. Phototaxis was scored after 48 hrs and images were recorded using brightfield microscopy.

### Differentiation and development

To follow differentiation and development, exponentially grown axenic cells (1–2 × 10^7^) were washed 1–2 times in KK2 and spread onto KK2 agar or nitrocellulose filters (Millipore) presoaked in KK2. Plates were incubated at 21/22°C for 48 hrs. Images were recorded using brightfield microscopy.

## Authors' contributions

**MZ: **Performed all published experiments, drafted original manuscript

**JK: **Established diploid techniques, made initial mutants and constructs

**KF: **Generated and tested anti-p21 antibodies

**RHI: **Supervised and discussed all experiments, edited manuscript

All authors have read and approved the final manuscript.

## Supplementary Material

Additional file 1 **Comparison of Arp2 from multiple eukaryote model systems. **Sequence alignment of Arp2 from *E. histolytica *(Eh), *D. discoideum *(Dd), *Caenorhabditis elegans *(Ce), *Drosophila melanogaster *(Dm), Mouse (Ms), Human (Hm) and *Saccharomyces cerevisiae *(Sc). Dark shading represents identical residues. Light shading represents conserved residues. Dashes are incorporated to optimise alignment. Click here for file

Additional file 2 **Scheme for the generation of a heterozygous ARP2 knock-out. (A) **The site of the blasticidin insertion is indicated, as are the locations of the PCR primers used for screening. **(B) **PCR screen for ARP2 disruption, showing markers (lane 1), DJK45 (lane 2), a random integrant (lane 3) and the parental DIR1 strain (lane 4). Successful disruption gives a product of 1.4 Kb whereas no product is generated with either wild-type or random integrant cells. Click here for file
