# Supplementary figures and images for: Replacement of the essential Dictyostelium Arp2 gene by its Entamoeba homologue using parasexual genetics
Source: BMC Genet. 2007 Jun 6;8:28. doi: 10.1186/1471-2156-8-28 (PMC1904233; doi:10.1186/1471-2156-8-28)

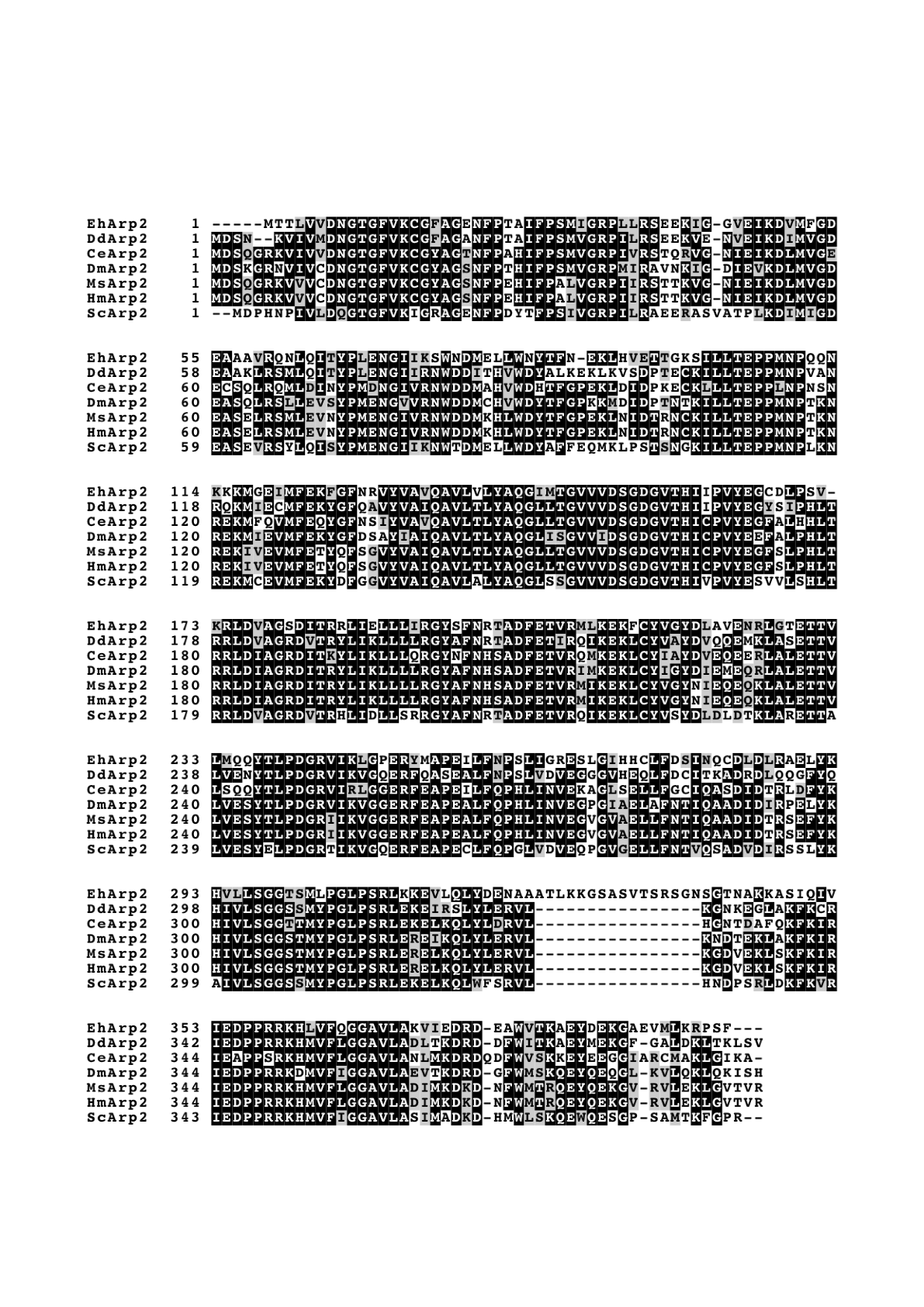

Supplement: Additional file 1 — Comparison of Arp2 from multiple eukaryote model systems. Sequence alignment of Arp2 from E. histolytica (Eh), D. discoideum (Dd), Caenorhabditis elegans (Ce), Drosophila melanogaster (Dm), Mouse (Ms), Human (Hm) and Saccharomyces cerevisiae (Sc). Dark shading represents identical residues. Light shading represents conserved residues. Dashes are incorporated to optimise alignment. [file 1471-2156-8-28-S1.png]
